# Halotolerant Rhizobacteria Promote Growth and Enhance Salinity Tolerance in Peanut

**DOI:** 10.3389/fmicb.2016.01600

**Published:** 2016-10-13

**Authors:** Sandeep Sharma, Jayant Kulkarni, Bhavanath Jha

**Affiliations:** ^1^Central Salt and Marine Chemicals Research Institute, CSIRBhavnagar, India; ^2^Academy of Scientific and Innovative Research, CSIRNew Delhi, India

**Keywords:** halotolerance, plant growth promoting rhizobacteria (PGPR), salinity stress, ion homeostasis, reactive oxygen species, *Arthrocnemum*, IAA production, acetylene reduction

## Abstract

Use of Plant growth promoting rhizobacteria (PGPR) is a promising strategy to improve the crop production under optimal or sub-optimal conditions. In the present study, five diazotrophic salt tolerant bacteria were isolated from the roots of a halophyte, *Arthrocnemum indicum*. The isolates were partially characterized *in vitro* for plant growth promoting traits and evaluated for their potential to promote growth and enhanced salt tolerance in peanut. The 16S rRNA gene sequence homology indicated that these bacterial isolates belong to the genera, *Klebsiella, Pseudomonas, Agrobacterium*, and *Ochrobactrum*. All isolates were *nifH* positive and able to produce indole -3-acetic acid (ranging from 11.5 to 19.1 μg ml^−1^). The isolates showed phosphate solubilisation activity (ranging from 1.4 to 55.6 μg phosphate /mg dry weight), 1-aminocyclopropane-1-carboxylate deaminase activity (0.1 to 0.31 μmol α-kB/μg protein/h) and were capable of reducing acetylene in acetylene reduction assay (ranging from 0.95 to 1.8 μmol C_2_H_4_ mg protein/h). These isolates successfully colonized the peanut roots and were capable of promoting the growth under non-stress condition. A significant increase in total nitrogen (N) content (up to 76%) was observed over the non-inoculated control. All isolates showed tolerance to NaCl ranging from 4 to 8% in nutrient broth medium. Under salt stress, inoculated peanut seedlings maintained ion homeostasis, accumulated less reactive oxygen species (ROS) and showed enhanced growth compared to non-inoculated seedlings. Overall, the present study has characterized several potential bacterial strains that showed an enhanced growth promotion effect on peanut under control as well as saline conditions. The results show the possibility to reduce chemical fertilizer inputs and may promote the use of bio-inoculants.

## Introduction

Peanut (*Arachis hypogaea* L) is an important cash crop of the leguminous family, grown in most of the arid, and semi-arid regions. Worldwide, peanut is cultivated on 26.4 million ha with a total annual production of 39.46 million metric tons (FAO, 2012; Sarkar et al., 2014). The production of peanut has shown to be repeatedly hampered by various abiotic stresses, such as high salt and drought, which ultimately, lead to severe loss in yield. Peanut being moderately salt tolerant, suffers massively by salinity stress due to its growth habitat in arid or semi-arid regions (Tanji and Kielen, 2002; Sun et al., 2013; Tiwari et al., 2015). Salinity stress has detrimental effects on almost every aspect of peanut growth and development including seed germination, early seedling establishment, photosynthesis, pod formation, total biomass, and finally, on yield production (Salwa et al., 2010; Qin et al., 2011; El-Akhal et al., 2013). It is, therefore, necessary to improve the salinity tolerance of peanut to minimize the yield loss.

Several approaches, including traditional breeding, and genetic engineering have been used to improve the salinity tolerance of peanut. However, such interventions have low success rate, mainly due to complexity of salinity tolerance and narrow genetic variability among germplasm accessions (Krishna et al., 2015). Use of bacterial inoculation, in particular, plant growth promoting rhizobacteria (PGPR), is effective and eco-friendly to improve plant stress tolerance. Several reports have shown that PGPR effectively improve growth of a wide range of agricultural crops under environmental stress conditions (Bacilio et al., 2004; Mayak et al., 2004b; Yuwono et al., 2005; Jha et al., 2009; Nabti et al., 2010; Ji et al., 2014; Islam S. et al., 2015; Majeed et al., 2015; Rolli et al., 2015; Timmusk et al., 2015; Zahid et al., 2015). In addition, the ability of PGPR to serve as bio-fertilizer or phyto-stimulator helps in maintaining the soil fertility, thereby providing a promising alternative to chemical fertilizers and pesticides for the sustainable agriculture (Majeed et al., 2015).

PGPR are free-living soil microbes that colonize roots and stimulate plant growth (Baldani et al., 1997; Schmid et al., 2009). A number of mechanisms are involved in plant growth promotion by PGPR. These include acquisition of nutrients, fixation of atmospheric nitrogen (N), phosphorus (P) solubilization, siderophore production, hydrocyanic (HCN) production, modulation of plant hormone and antagonistic action against biotic pathogens. The N-fixation, P- availability and the hormonal response have direct involvement in plant growth promotion; however, other mechanisms indirectly support the plant growth (Gontia et al., 2011; Bhattacharyya and Jha, 2012; Glick, 2012; Estrada et al., 2013; Vacheron et al., 2013; Abd El Daim et al., 2014).

The PGPR use several mechanisms to protect the plant growth under various abiotic stresses. Rhizobacteria activate plant antioxidant defense machinery by upregulating the activity of key enzymes, such as superoxide dismutase (SOD), peroxidase and catalase that scavenges overproducing reactive oxygen species (ROS) and protect the plants from salt toxicity (Jha and Subramanian, 2014; Islam F. et al., 2015). Under salinity stress, PGPR-inoculated plants gain increased efficiency to uptake selective ions to maintain a higher K^+^/Na^+^ ratio than non-inoculated plants (Shukla et al., 2012; Islam F. et al., 2015). PGPR producing 1-aminocyclopropane-1-carboxylate (ACC) deaminase, an enzyme that converts plant ethylene precursor ACC to ammonia and α-ketobutyrate. This metabolic event decreases plant ethylene level which in turn resumes plant growth under abiotic stresses (Glick, 2014; Singh et al., 2015). PGPR-inoculated plants have also been shown to have a change in root architecture. This may be due to increased indole -3-acetic acid (IAA) level that enables plant to uptake more nutrients under salinity stress condition (Vacheron et al., 2013; Goswami et al., 2014). A number of rhizobacteria emit stress-related volatile compounds that enhance plant biomass and survival under severe drought stress (Timmusk et al., 2014). Recently, Rolli et al. (2015) studied eight osmotolerant bacterial isolates that showed stress-dependent plant growth promoting activities and were capable in improving grapevine growth under drought stress. A mutant strain of *Paenibacillus polymyxa*, A26Δ*sfp*, that had inactivation of A26 Sfp-type 4′-phosphopantetheinyl transferase enzyme (Sfp-type PPTase) showed greatly enhanced biofilm activity and produced two times higher plant survival and three times increased wheat biomass under drought stress (Timmusk et al., 2015). This mutant strain is also an efficient antagonistic agent against *Fusarium* spp. causing *Fusarium* head blight disease in cereals (Abd El Daim et al., 2014).

Halophytes are extremely salt tolerant plants which usually grow near a coastal area where no cultivation occurs. The rhizosphere of halophytic plants represent ideal source for isolation of various groups of salt tolerant rhizobacteria that could enhance the growth of different crops under salinity stress (Shukla et al., 2012; Bharti et al., 2013; Ramadoss et al., 2013; Goswami et al., 2014; Jha et al., 2015). Efforts have been made to isolate few halotolerant bacteria that confers salt tolerance to agricultural crops. Examples of such bacteria are *Brachybacterium* sp. (Jha et al., 2012; Shukla et al., 2012), *B. licheniformis* (Goswami et al., 2014), *Exiguobacterium oxidotolerans* (Bharti et al., 2013), *Pseudomonas* sp. (Rosenberg, 1983; Egamberdieva et al., 2015), and *Hallobacillus* sp. (Ramadoss et al., 2013). However, scant information is available for potential halotolerant PGPR that promotes salinity tolerance to peanut.

*Arthrocnemum indicum* (Chenopodiaceae family) is a stem succulent perennial halophytic shrub, generally found in tropical salt marshes that are frequently inundated with seawater. It has high antioxidant and anti-radical activity (Boulaaba et al., 2013). Moreover, it is a potential source of anti-cancer molecules, used in the treatment of snakebites and scorpion stings thus, has medicinal significance (Boulaaba et al., 2013). The present study was undertaken to isolate bacterial strains from the roots of *A. indicum*, their characterization and efficacy to test their ability to promote growth and tolerate salinity of peanut. Our results indicate that the *A. indicum* roots have several bacteria that enhances growth and confer salt tolerance to peanut seedlings.

## Materials and methods

### Isolation of bacterial isolates

Bacterial isolation from the roots of *Arthrocnemum indicum* was performed according to Jha et al. (2012). Briefly, roots (0.5 g fresh weight) were washed thoroughly, homogenized with 0.5X PBS (9.5 ml), serially diluted and grown on nitrogen-free semisolid NFb medium containing up to 4% NaCl and malate as a sole carbon source at 30°C. Bacteria were subcultured two more times. Finally, bacteria were streaked onto solid NFb medium with 20 mg l^−1^ of yeast extract. Single, distinct colonies were analyzed for pellicle formation and purified further.

### Physiological and biochemical characterization

Biochemical tests for utilization of different carbon sources, such as sugars, organic acids, and amino acids were performed using BIOLOG identification system (BIOLOG Microstation™, Biolog Inc., Hayward, CA). GEN III MicroPlate™ analyses 94 phenotypic tests: 71 carbon source and 23 chemical sensitivity assays were inoculated according to the BIOLOG manufacturer's directions. Plates were covered and incubated at 30°C for 24 h. Measurement of MicroPlate were taken with a BIOLOG microplate reader. The enzyme activities (Amylase, Gelatinase, pectinase, lipase, Catalase) were measured as described previously by Jha et al. (2012). Motility tests for PGPR were performed using Motility Test Medium (Himedia, Mumbai) (Camper et al., 1993). For ammonia production, isolates were cultured in peptone broth and incubated at 30°C for 48–72 h. Following incubation, Nessler's reagent (Sigma, USA) was used to detect brown to yellow color formation as described previously by Jha et al. (2012).

### Molecular characterization of bacterial isolates

Genomic DNA was isolated from bacterial strains using standard protocol (Sambrook et al., 1989). The universal primers 27F (5′- AGAGTTTGATCMTGGCTCAG-3′) and 1492R (5′- TACGGYTACCTTGTTACGACT-3′) were used to amplify 16S gene sequences by PCR (Lane, 1991). Amplified gene sequences were gel purified using QIAquick gel extraction kit (Qiagen, Germany) and sequenced. Sequence comparison was then performed with obtained 16S rRNA gene sequences against sequences in NCBI and Silva rRNA database (https://www.arb-silva.de/). For *nifH* screening, a 360-bp fragment was amplified using PolF and PolR primers (Poly et al., 2001) and the amplified DNA fragment was purified and sequenced. Phylogenetic analysis of 16S rRNA and nifH amino acid sequences were performed using MEGA version 6. The Neighbour- Joining method (Saitou and Nei, 1987) and bootstrap analysis (Felsenstein, 1985) were performed using 1000 bootstrap replications and evolutionary distance were computed with the Maximum Likelihood method (Tamura et al., 2004).

### Bioassays for plant growth promoting traits

#### Biological nitrogen fixation (BNF)

BNF was measured by acetylene reduction/ethylene production assay (ARA) as described previously by Majeed et al. (2015). Briefly, pure bacterial cultures were inoculated in 20 ml airtight vials containing 5 ml semisolid nitrogen free NFb medium and grown for 48 h at 28°C. Following pellicle formation, 10% (v/v) acetylene gas was injected into the vials, which were incubated for 16 h at 30°C. Samples were then analyzed by gas chromatography (GC) (Shimadzu, Japan) using RTX5 column (Restek, USA) and a H_2_-flame ionization detector (FID). The peaks of acetylene and ethylene were also confirmed by GC-MS (Shimadzu, Japan). All the experiments were performed in triplicates.

#### Indole-3-acetic acid (IAA)

Indole-3-Acetic Acid (IAA) production of bacteria was determined by colorimetric method as described by Patten and Glick (2002). 50 μl of overnight grown cultures were transferred into 10 ml of nitrogen-free NFb medium supplemented the presence and absence of 0.05% L-tryptophan. Cultures were then incubated at 28°C with continue shaking at 180 rpm for 48 h. The density of each culture was measured spectrophotometrically at 600 nm, and then the culture was centrifuged at 13000 rpm for 5 min. One ml aliquot of resulting supernatant was then mixed with 2 ml Salkowski's reagent and incubated in the dark at room temperature for 20 min. IAA production was confirmed by observing pink color formation, and the absorbance was measured at 530 nm. A known IAA standard was used to determine IAA concentrations. *A. brasilense* strain Sp7 was used as a positive control in the present study.

#### Phosphate (P) solubilisation

The bacterial cultures were inoculated into 10 ml Pikovskaya broth (Himedia, Mumbai) containing tri-calcium phosphates and incubated at 28°C for 72 h. Following incubation, 1 ml of culture was withdrawn and the cell-free supernatant was mixed with 4 ml of Chen reagent and incubated at 37°C for 1 h 30 min. The reaction product, phosphomolybdate, was then determined by measuring OD at 619 nm after adjustment to a final volume of 8 ml (Goldstein, 1986).

#### Assays for other growth promoting traits

ACC deaminase activity was determined as described previously by Penrose and Glick (2003). The formation of orange-yellowish halos surrounding bacterial colonies on CAS agar plates after 48 h of incubation at 30°C indicated siderophore production (Schwyn and Neilands, 1987). The zinc solubilisation assay was performed according to Fasim et al. (2002) and HCN production was carried out according to the method of Lorck (1948).

#### Bacterial growth promotion effects on peanut

Peanut cv. GG 20 seeds were surface sterilized with 2% sodium hypochlorite and rinsed 4–5 times with sterilized double-distilled water. Seeds of uniform size were placed in sterile cotton (soaked in ½MS medium) in a tissue culture bottle and kept in the dark for 2 days before transfer to a growth chamber. The growth conditions were set at 26°C and 16/8 h light/dark cycle (350 μmol m^−2^ s-^1^ light intensity). After a week, seedlings were transferred to hydroponics containing half MS medium (without sucrose and nitrogen source) with and without bacterial inoculum and growth parameters were measured after 21 days. For inoculation, single bacterial colony was inoculated in 5 ml of half DYGS medium (Kirchhof et al., 2001) and kept at 30°C on shaker incubator (180 rpm) for 24 h. The bacterial culture was adjusted to OD 0.6 that correspond to approximately 10^8^ cells/ml. Five ml of this bacterial culture was added to 250 ml of ½MS medium. The hydroponic medium was changed weekly. The shoot and root samples were dried separated and used for dry weight measurement.

In soil pot experiment, peanut seeds were pre-inoculated with bacterial inoculum (MBE02, MBE03, and positive strain) and kept in dark for 2 d and the seedlings were transferred to the pots (3 kg soil capacity, three seedlings/pot) filled with garden soil. Soil samples were autoclaved twice before the experiment. One ml of bacterial culture (OD 0.6) was placed at the base of the seedlings after 3 weeks of germination. Seedlings without inoculation served as control. The plants were harvested after 60 d for root and shoot biomass measurement. For field trial, seedlings were grown in 2 × 4 m plots with the spacing of 30 cm between rows and 15 cm between plants. The bacterial treatments were given in a similar way as described for greenhouse experiment. The treatments, including control, were replicated thrice in randomized complete block design. N and P were applied at a half rate of recommended dose. Standard agronomical practices were followed to maintain the plots. The plant biomass was measured after 40 days of germination.

For salt treatment, 12 d old peanut seedlings, grown in half MS medium were treated with 100 mM NaCl with or without bacterial inoculum and growth were measured after 18 d.

### Root colonization

Root colonization assay was performed as described previously by Majeed et al. (2015). Root samples were collected from bacteria inoculated peanut seedlings at 10th and 20th day post-inoculation. Root surface was thoroughly washed with running tap water to remove weakly bound cells. After washing, the plant roots were blotted dry, weighed and 1 g was homogenized with 10 ml autoclaved distilled water using sterile mortar and pestle. Serial dilutions (up to 10^−7^) were then plated on ½DYGS medium plates and incubated at 30°C for 24–48 h. Colony forming unit (CFU) per gram root was determined as described previously by Islam S. et al. (2015). Three replications were used for each treatment and the experiment was repeated three times.

### Ion analysis and nitrogen measurement

For ion analysis, 0.5 gram of dried shoot and root was digested with 5 ml of perchloric acid and nitric acid solution (3:1). The solution was heated on a hot plate and diluted to 25 ml with autoclaved deionized water and filtered through a 0.22 μm filter. Na^+^, K^+^, and Ca^2+^ content was measured using an inductively coupled plasma optical emission spectroscopy (ICP). For nitrogen measurement, dried shoot and root sample were crushed into fine powder and analyzed using an Elemental analyzer (Elementar, Vario Micro Cube, Germany).

### *In vivo* localization of peroxide and superoxide radicals

The upper most leaves of 3–4 different plants were combined and immersed into 0.5 mg/ml nitroblue tetrazolium (NBT, Sigma) solution in 10 mM phosphate buffer (pH 7.8) for 2 h in the dark for superoxide detection. Whereas, for H_2_O_2_ visualization, leaves were stained with 3,3'-diaminobenzidine tetrahydrochloride (DAB) solution (in 10 mM phosphate buffer, pH 3.8) for 6 h in the dark. Thereafter, for both the assays, samples were exposed to light and treated with destaining solution (ethanol: acetic acid: glycerol; 3:1:1 v/v) for 15 min at 95°C, and then rehydrated in 40% glycerol.

### Salt sensitivity test of PGPR

Salt tolerance of the PGPR in the presence of a nitrogen source was observed in nutrient broth (NB) medium supplemented with 1–20% NaCl. Fresh bacterial cultures were inoculated in 5 ml NB medium and incubated at 30°C with constant shaking at 180 rpm for 24 h. Bacterial growth was then determined by measuring the OD at 600 nm. Additionally, salt tolerance of PGPR in nitrogen-free semisolid NFb was also tested. Bacteria were enriched in semisolid NFb containing up to 4% NaCl (w/v) for 5–7 days at 30°C (Jha et al., 2012).

### RNA isolation, cDNA synthesis and real-time PCR

Total RNA was isolated from peanut seedlings using Tri reagent (Sigma, USA) according to the manufacturer's instructions. RNA was quantified using a ND-1000 spectrophotometer (NanoDrop), and 0.5 to 1 μg of RNA was reverse transcribed by using ImProm-II™ reverse transcription kit (Promega, USA). For quantitative PCR analysis, 5–10 fold diluted cDNA was used in a reaction mixture with QuantiFast SYBR Green PCR reaction kit (Qiagen, Germany) and real time quantification was performed using Real-Time iQ5 Cycler (Bio-Rad, USA). Two to three biological samples for each treatment were processed in triplicates. *Ah-actin* was used as an internal control and relative fold change was determined by 2^−ΔΔCt^ method (Livak and Schmittgen, 2001). The real time primers used in the present study were previously reported by Yang et al. (2013) and Tiwari et al. (2015) and are given in Supplementary Table 4.

### Statistical analysis

Data analysis was performed by using IBM SPSS statistics 19. Means of different treatments were compared by one way ANOVA using Student-Newman-Keuls test (SNK test) at 5% probability level (*P* < 0.05). The real time data was analyzed by one way ANOVA using Dunnett's test. Wherever needed, means among different treatments were compared by *t*-test (*P* < 0.05).

## Results

### Enrichment of bacterial isolates and *nifH* sequence analysis

Five different bacterial isolates (MBE01, MBE02, MBE03, MBE04, and MBE05) obtained from roots of *A. indicum* were capable to grow on nitrogen free semi-solid NFb medium containing up to 4% NaCl and malate as sole carbon source. The growth of bacteria under these conditions indicated the ability of bacterial isolates to fix atmospheric N, which was further confirmed by amplification of *nifH* gene and the acetylene reduction assay (see below). The sequencing of the amplified products showed similarity with *nifH* gene. The phylogenetic tree based on NifH amino acid sequences was constructed (Supplementary Figure 1) and the sequences were submitted to Genbank under accession numbers KX215161, KX215162, KX215163, KX215164, and KX215165 for isolates MBE01, MBE02, MBE03, MBE04, and MBE05, respectively.

### Biochemical and molecular characterization of bacterial isolates

Biochemical analysis revealed all the isolates to be gram-negative, with an ability to exhibit catalase activity and produced ammonia. Except MBE02, rest of the isolates were positive in motility test (Table 1 and Supplementary Table 1). Other biochemical and physiological parameters were analyzed and are summarized in Supplementary Table 1.

**Table 1:** **Biochemical and molecular analysis of different bacterial isolates**.

**Isolate code**	**IAA μg ml^−1^**	**P-solubilization^a^**	ACC deaminase activity^*^	**Catalase**	**ARA μmol C_2_H_4_ mg protein/h**	**Identification based on 16 s rRNA sequencing**
MBE01	16.9 ± 0.14	26.5 ± 0.64	0.12	+	1.68	*Agrobacierium tumefaciens*
MBE02	19.1 ± 0.45	55.6 ± 4.2	0.17	+	1.8	*Klebsiella* sp.
MBE03	11.5 ± 0.31	26.2 ± 1.0	0.10	+	1.41	*Ochrobactrum anthropi*
MBE04	11.5 ± 0.43	1.40 ± 0.46	0.31	+	0.95	*Pseudomonas stutzeri*
MBE05	16.3 ± 3.15	18.9 ± 0.67	0.19	+	1.3	*Pseudomonas* sp.
Sp7	34.1 ± 1.51	59.2 ± 7.8	0.25	+	1.29	

*“+” corresponds to a positive response. Data are means ± SE (n = 3–5). Experiments were repeated twice observing the same trend. A. brasilense strain Sp7 was used as positive control*.

a *μg phosphate/mg dry weight*.

* *μmol α-ketobutyrate μg protein^-1^ h^-1^*.

Sequence analysis of 16S rRNA gene revealed that isolates MBE01, MBE02, MBE03, MBE04, MBE05 to share sequence identity with *A. tumefaciens* (98%), *Klebsiella* sp. (100%), *Ochrobactrum anthropi* (99%), *P. stutzeri* (99%), and *Pseudomonas* sp. (99%), respectively. A phylogenetic tree was constructed based on the 16S rRNA sequences shows the taxonomic positions of the isolates is shown in (Supplementary Figure 2). The sequences were submitted to Genbank under accession numbers KX083679, KX083680, KX083681, KX083682, and KX083683 for the isolates MBE01 to MBE05, respectively.

In a comparison between *nifH* and 16S rRNA tree, it was observed that NifH protein of MBE01 was highly related to *Azospirillum* species; however, it had similarity with *A. tumefaciens* in 16S tree. Similarly, MBE03 fall in the group of *Pseudomonas* with MBE04 and MBE05 but it belong to *Ochrobactrum* in 16S tree (Supplementary Figures 1, 2). The incongruence of these dendrograms as regards the position of MBE01 and MBE03 suggests the possibility of lateral transfer of *nifH* (Haukka et al., 1998).

### Estimation of plant growth promoting traits

Plant growth promoting traits, such as IAA production, P-solubilization, acetylene reduction activity, siderophore, and HCN production, were analyzed to evaluate the putative plant growth promoting activities of isolates. Results obtained indicated all of the five isolates were able to synthesize IAA. MBE03 and MBE04 had the lowest ability to produce IAA; however, MBE02 had highest IAA concentration followed by MBE01 (Table 1). None of the bacterial isolates were able to synthesize IAA in the absence of external tryptophan (data not shown). *A. brasilense* Sp7, used as a positive control, showed higher IAA level (~1.7 to 2.9-fold) than other isolates. This is consistent with previously published reports where *Azospirillum* strains were shown to produce high IAA content (Akbari et al., 2007).

MBE04 and MBE05 showed lowest; however, MBE02 had highest P-solubilization activity followed by MBE01. All bacteria isolates were positive for nitrogenase activity where MBE02 and MBE01 showed maximum ARA. The highest ACC deaminase activity was observed for MBE04 followed by MBE05 isolate (Table 1). The positive control values obtained from these assays are given in Table 1. Other PGPR traits, such as siderophore and HCN production and zinc solubilization were, also, measured and shown in (Supplementary Table 2).

### PGPR promotes peanut growth under non-stress condition

The efficacy of bacterial isolates as PGPR in growth promotion of peanut seedlings was evaluated. The results indicated that all isolates including positive control significantly increased the growth of peanut compared to control (SNK test *P* < 0.05; Table 2). The relative increase in shoot and root length varied between 14–70% and 12.9–36%, respectively. Improvement in shoot and root biomass ranged between 21–44% and 36–64% over the non-inoculated control, respectively. MBE02 exhibited highest desirable trait for plant biomass (Table 2).

**Table 2:** **Effect of PGPR treatment on various growth parameters of peanut under non-stress condition**.

	**Shoot length (cm)**	**Shoot fresh weight (g)**	**Shoot dryweight (g)**	**Root length (cm)**	**Root fresh weight (g)**	**Root dry weight(g)**	**Total N (mg g^1^)**
Non-ino	11.2 ± 0.3^a^	2.89 ± 0.07^a^	0.47 ± 0.026^a^	7.77 ± 0.37^a^	0.76 ± .023^a^	0.067 ± .003^a^	21 ± 1.8^a^
MBE01	15.27 ± 0.52^c^	3.44 ± 0.23^b^	0.57 ± 0.02^b, c^	8.78 ± 0.84^ab^	0.88 ± 0.06^c^	0.10 ± .003^b, c^	37.1 ± 2.34^b^
MBE02	16.65 ± 0.8^c^	3.72 ± 0.14^b^	0.68 ± 0.02^c^	10.23 ± 0.44^b^	1.14 ± 0.05^b, cd^	0.11 ± 0.005^c^	29.2 ± 0.94^b^
MBE03	19.05 ± 0.65^d^	4.34 ± 0.20^c^	0.59 ± 0.013^b, c^	10.6 ± 0.37^b^	1.07 ± 0.05	0.095 ± 0.005^b, c^	30.9 ± 0.64^b^
MBE04	12.8 ± 0.46^ab^	3.68 ± 0.18^b^	0.59 ± 0.017^bc^	10.1 ± .61^b^	0.93 ± 0.04^a, b, c^	0.10 ± 0.006^b, c^	27.4 ± 1.96^b^
MBE05	13.07 ± 0.47^ab^	3.76 ± 0.15^b^	0.57 ± 0.03^b, c^	10.07 ± 0.6^b^	0.98 ± 0.05^b, c^	0.091 ± 0.003^b^	29.9 ± 2.07^b^
Sp7	14.7 ± 0.4^b, c^	3.56 ± 0.18^b^	0.57 ± 0.02^b^	9.9 ± 0.29^b^	0.99 ± 0.05^b, c^	0.09 ± 0.003^b, c^	29.3 ± 1.4l^b^

*Data are means ± SE and combined from 2 to 3 independent experiment (n = 10–20). Different letter indicates significant difference between data of the same column and calculated by one way ANOVA SNK test (P < 0.05). A. brasilense strain Sp7 served as positive control*.

N contents were measured in the shoot and the root of bacteria inoculated and non-inoculated peanut seedlings and shown as total plant N in Table 2. A significant increase in the N content was observed for inoculated seedlings, values varied between 27.4±1.96 and 37.1±3.19 mg g^−1^ as compared to 21.0±1.8 mg g^−1^ for control. The isolate MBE01 caused highest N content.

**Figure 1:**
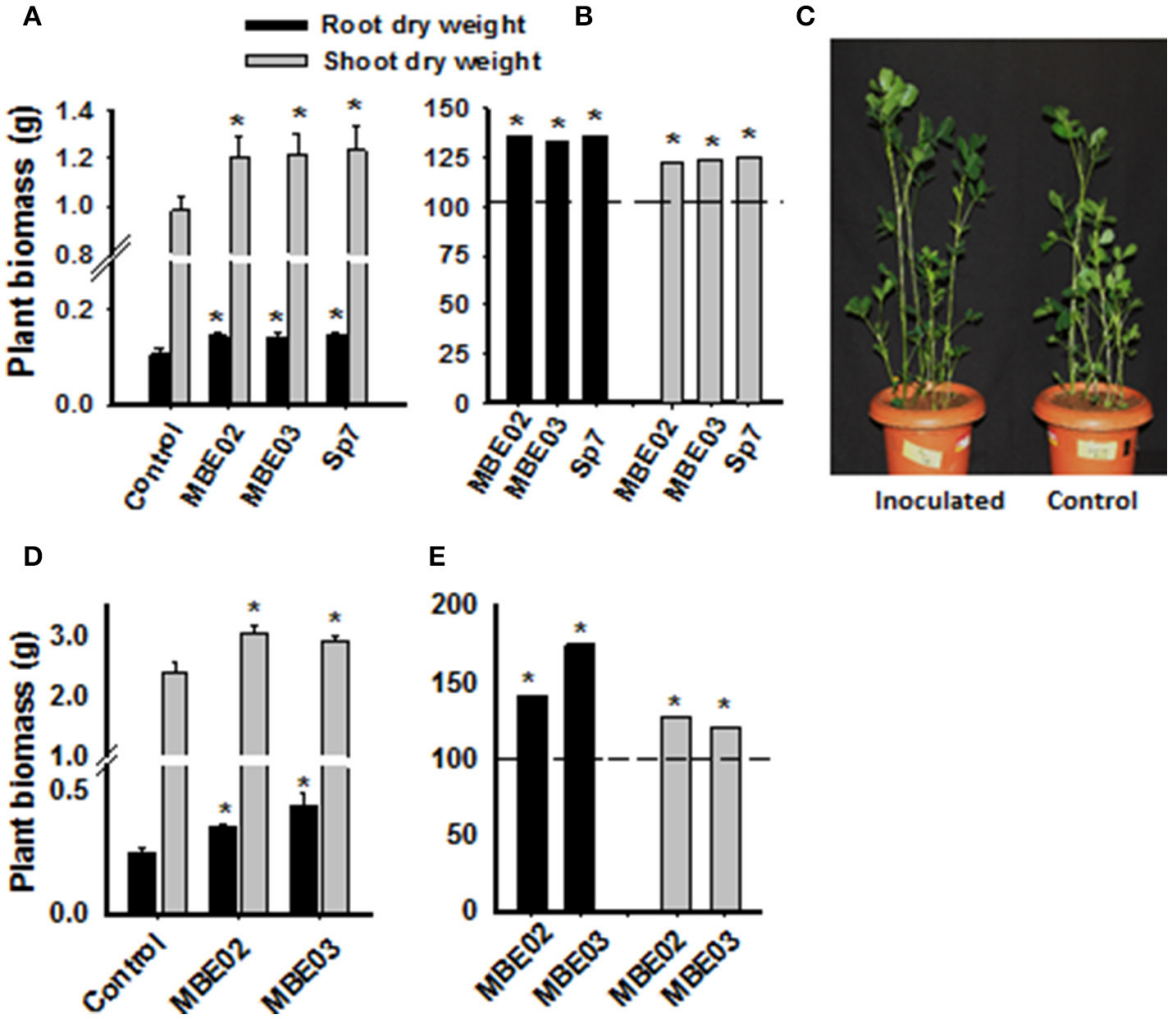
**Effect of PGPR on growth promotion of peanut plants under greenhouse and field conditions. (A)** Seeds of peanut (GG20) were pre-treated with bacterial inoculation and then transferred into the soil pot and measurement was taken after 60 days of germination. Data are means ± SE (*n* = 10–15). Significant differences are shown by Asterisks (^*^) calculated by *t*-test (*P* < 0.05). **(B)** Data from **(A)** is shown as % increase as compared to the non-inoculated control represented as a dash line. *A. brasilense* Sp7 was used as positive control. **(C)** A representative pot of inoculated and non-inoculated peanut seedlings of 60 days after germination. **(D)** Pre-inoculated peanut seeds were grown in field plots and measurement was taken after 40 days of germination. Data are means ± SE (*n* = 14–15). Significant differences are shown by Asterisks (^*^) calculated by *t*-test (*P* < 0.05). **(E)** Data from **(D)** is shown as % increase as compared to the non-inoculated control represented by dash line.

The growth promotion ability of two selected isolates (MBE02, MBE03) and the positive control were tested in a pot experiment under greenhouse conditions using sterile soil. All inoculated plants had increased biomass as compared to non-inoculated control (Figures 1A–C). Similar results were obtained for peanut cultivated in field plots (Figures 1D,E).

Plate count for root colonization efficiency of bacterial isolates revealed that isolates were able to colonize the roots, among which, highest colonization rate was observed for MBE05 at 10th and 20th day after inoculation, followed by MBE04. (Supplementary Figure 3).

### Halotolerant PGPR enhances salt tolerance in peanut

**Figure 2:**
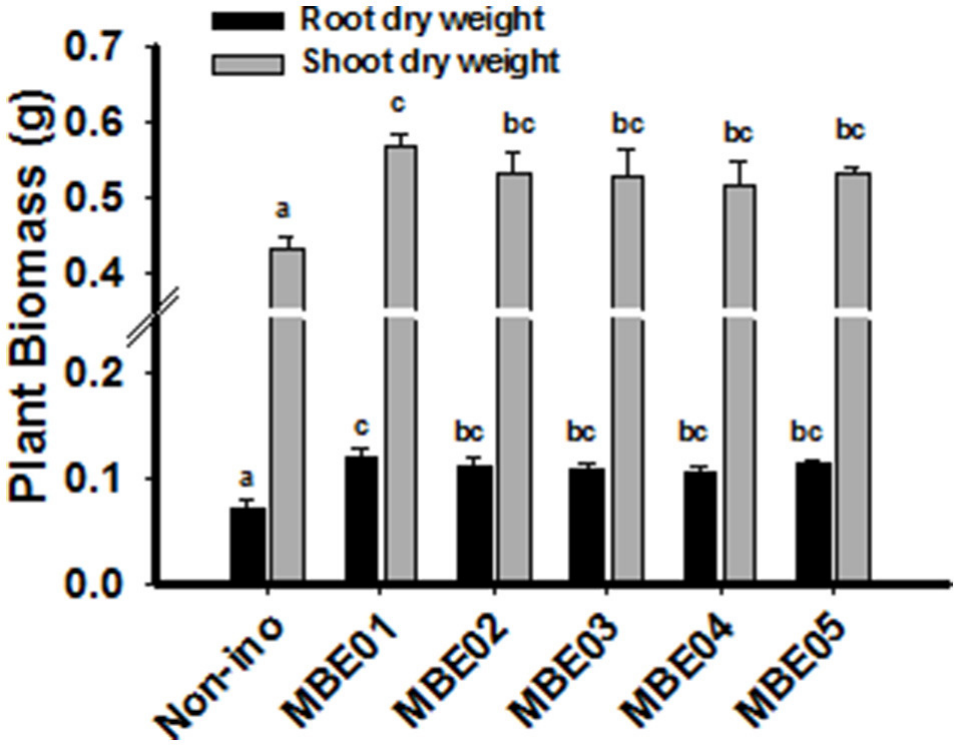
**Effect of PGPR treatment on salt tolerance of peanut seedlings**. 12 d old peanut seedlings were treated with 100 mM NaCl in the presence or absence of bacterial inoculation and plant biomass was determined 18 days later. Data are means ± SE and combined from two independent experiments (*n* = 15–20). Different letters indicate significant difference calculated by one way ANOVA SNK test (*P* < 0.05).

Since bacterial isolates originate from halophytic plant, salt tolerance capacity of the isolates were evaluated by growing them in nutrient broth (NB) medium supplemented with different concentrations of NaCl. Maximum NaCl tolerance was shown by MBE02 (up to 8%); the rest failed to exhibit the same trait (Supplementary Table 3). To determine if bacterial isolates could enhance salt tolerance in peanut seedlings, 12 d old seedlings in the presence of 100 mM NaCl and PGPR were grown and growth measurement was monitored 18 days. All isolates were found to significantly improve the seedling growth over the control (Figure 2). Relative increase in shoot and root biomass for PGPR treated seedlings ranged between 19–31% and 45–64%, respectively. Other parameters, such as plant fresh weight and length were determined and are shown in Supplementary Figure 4.

Results obtained with estimation of intracellular ions with shoot and root of bacteria treated and non-treated peanut seedlings under salinity stress are summarized in Table 3. Seedlings inoculated with MBE01, MBE02, and MBE05 had significantly lower shoot Na^+^/K^+^ ratios in comparison to non-inoculated seedlings (SNK test *P* < 0.05). Rest of the isolates failed to exhibit similar effect. The shoot Ca^+^ content was, also, significantly higher in seedlings inoculated with all isolates but, not with the MBE01 (Table 3).

**Table 3:** **Ion analysis in the shoot and root of peanut seedlings subjected to 100 mM NaCl treatment up to 18 days**.

	**Shoot ion content (mg g^−1^ DW)**				**Root ion content (mg g^−1^ DW)**			
	**Ca^+^**	**Na^+^**	**K^+^**	**Na^+^/K^+^ ratio**	**Ca^+^**	**Na^+^**	**K^+^**	**Na^+^/K^+^ ratio**
Control	3.28 ± 0.50^d^	0.41 ± 0.008^c^	20.2 ± 1.52^b^	0.02 ± 0.01^d^	0.37 ± 0.08^a, b^	0.55 ± 0.15^c^	6.35 ± 1.37^a^	0.08 ± 0.01^c^
Non-inoSalt	1.21 ± 0.03^a^	14.2 ± 2.36^a^	11.3 ± 0.45^a^	1.27 ± 0.05^a^	0.14 ± 0.05^a^	2.41 ± 0.22^a^	5.94 ± 0.99^a^	0.43 ± 0.08^a^
MBE01	1.44 ± 0.31^a, b^	7.98 ± 0.965^ab^	13.70 ± 1.5^a^	0.60 ± 0.12^c^	0.15 ± 0.02^a^	1.43 ± 0.08^a^	6.20 ± 0.52^a^	0.23 ± 0.02^b^
MBE02	2.30 ± 0.34^b, c^	10.0 ± 1.65^a, b^	12.95 ± 0.47^a^	0.77 ± 0.05^b, c^	1.09 ± 0.2^c^	2.57 ± 0.24^a^	4.74 ± 0.52^a^	0.54 ± 0.03^a^
MBE03	1.96 ± 0.72^c^	12.9 ± 0.23^a^	12.90 ± 0.21^a^	1.0 ± 0.02^ab, c^	0.68 ± 0.24^b, c^	2.35 ± 0.3^a^	5.28 ± 0.42^a^	0.43 ± 0.02^a^
MBE04	2.23 ± 0.01^b, c^	13.81 ± 0.72^a^	11.17 ± 0.67^a^	1.23 ± 0.03^ab^	0.23 ± 0.1^a, b^	2.50 ± 0.17^b^	6.44 ± 0.36^a^	0.38 ± 0.01^a^
MBE05	2.21 ± 0.14^b, c^	10.75 ± 1.41^a^	12.6 ± 0.48^a^	0.85 ± 0.15^b, c^	0.31 ± 0.02^a^	2.88 ± 0.11^a^	6.12 ± 0.28^a^	0.47 ± 0.02^a^

*Data shown are means ± SE and combined from two independent experiments (n = 8-10). Different letter indicates significant difference calculated by one way ANOVA SNK test (P < 0.05)*.

Treatment of seedlings with MBE01 significantly lowered the root Na^+^/K^+^ ratio as compared to non-treated samples. However, other bacterial isolates failed to exhibit similar effect (Table 3). The root Ca^+^ was found to be altered when the seedlings were treated with MBE02 and MBE03.

**Figure 3:**
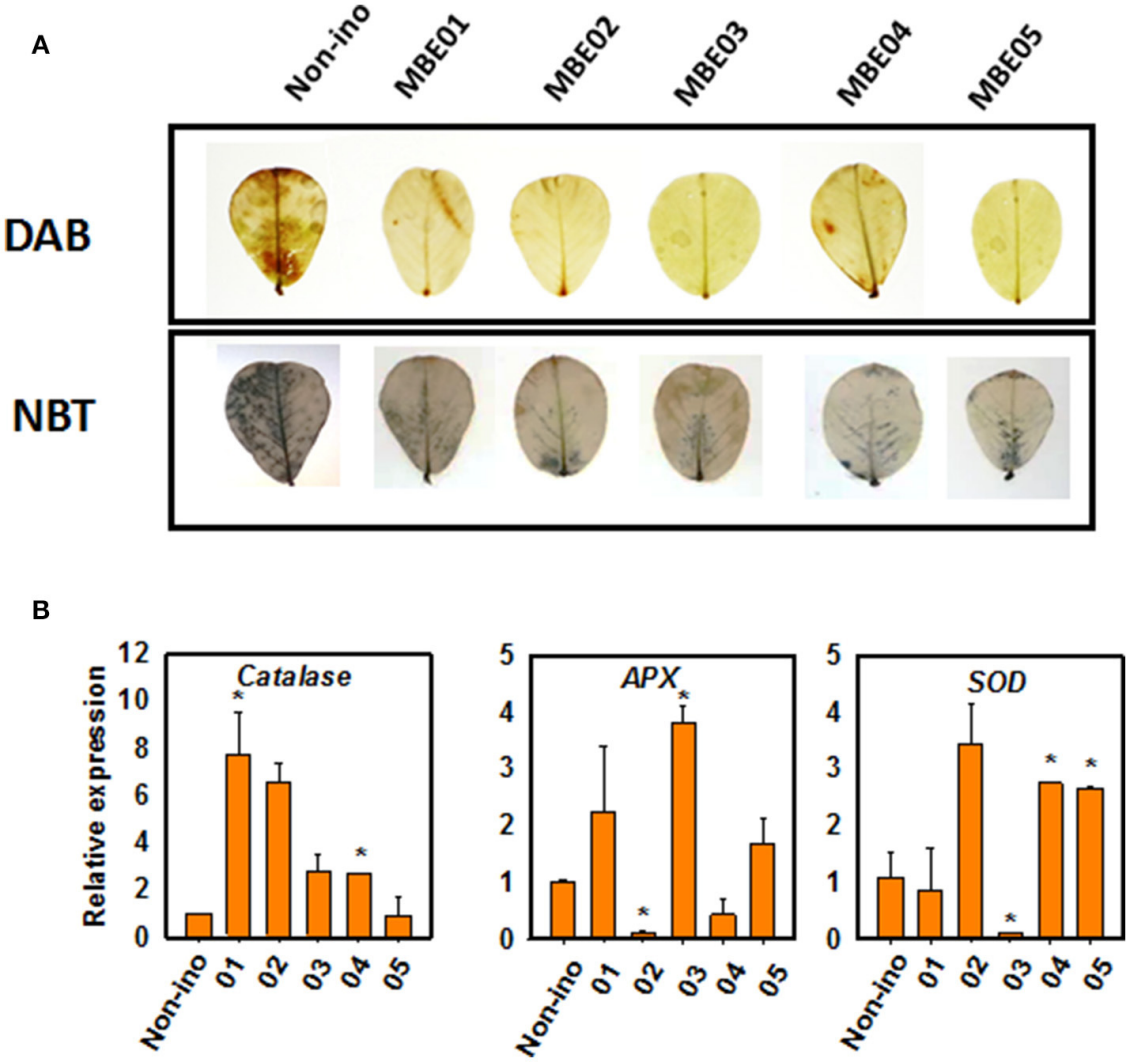
**ROS accumulation and antioxidant gene expression analysis in PGPR inoculated or non-inoculated salt treated peanut seedlings. (A)** DAB and NBT staining for *in vivo* localization of superoxide and hydrogen peroxide. The upper most leaves (4–5) from 3 to 4 different plants for each treatment was used and the picture of a representative leaf is shown here. **(B)** Quantitative expression analysis of antioxidant genes, ascorbate peroxidase (*APX*), catalase (*CAT*), and superoxide dismutase (*SOD*). Asterisks (^*^) indicate significant differences as analyzed by Dunnett's test (*P* < 0.05). The expression was quantified relative to non-inoculated salt treated seedlings.

We next determined the accumulation of superoxide and hydrogen peroxide by using nitroblue tetrazolium (NBT) and 3,3′-diaminobenzidine (DAB) staining. Salt treated seedlings in the absence of bacterial inoculum accumulated more reactive oxygen species (ROS) than the seedlings inoculated with PGPR (Figure 3A). This shows PGPR treatment might have led to differential regulation on the expression of antioxidant genes, such as ascorbate peroxidase (*APX*), catalase (*CAT*), and superoxide dismutase (*SOD*) (Figure 3B). Catalase levels were significantly induced by MBE01 and MBE04 but not by others. Expression of *APX* was significantly induced by MBE03 but decreased by MBE02. Isolates MBE04 and MBE05 induced expression of SOD whereas, the same was significantly reduced when peanut seedlings were treated with MBE03. The other isolates had no significant effect on SOD level (Figure 3B).

## Discussion

Soil salinity is a major obstacle for the production of agriculture crops growing in arid and semi-arid regions. Use of halophilic or salt tolerant PGPR is an effective approach that has been employed successfully in various crops to improve their growth and tolerance under salt stress condition. The halotolerant bacteria are able to withstand high salt concentration because of their capability to accumulate compatible osmolyte to maintain intracellular osmotic balance (Nabti et al., 2015). These bacteria are positive for multiple stress-related traits that may facilitate plants to survive under growth inhibitory levels of salt (Rohban et al., 2009; Siddikee et al., 2010; Bharti et al., 2013). In the present study, salt tolerant rhizobacteria were isolated from the roots of *A. indicum* and growth potential of these bacteria were determined under control and salinity stress. The isolates were tolerant to 4–8% NaCl in NB medium and 3–4% NaCl concentration in nitrogen free Nfb medium. They were gram negative, *nifH* positive and belong to the genera *Klebsiella, Pseudomonas, Agrobacterium*, and *Ochrobactrum*. *Pseudomonas* is the most commonly reported genera in PGPR and the isolates belonging to this genera have shown to be involved in conferring salinity tolerance in various crop species (Jha et al., 2012). Similar reports are available for salt tolerant *Agrobacterium* and *Klebsiella* (Shukla et al., 2012; Liu et al., 2016). However, *Ochrobactrum* has mostly been used in phytoremediation (Al-Mailem et al., 2010) and its role in enhancing salinity tolerance of crop plants has not been studied extensively. The present study has demonstrated that halotolerant *Ochrobactrum* improves peanut growth under salinity stress condition.

The present isolates showed response for ACC deaminase and catalase activity. PGPR with ACC deaminase activity have been known to protect plants against environmental stresses by reducing the ethylene levels (Mayak et al., 2004b; Singh et al., 2015). Similarly, catalase, present in most of the aerobic bacteria, helps in maintaining plant ROS levels during stress (Cowell et al., 1994). Our observations on the above traits are in consistent with previous reports where diverse halotolerant bacteria were found to exhibit both of the enzymatic activities to promote plant growth under environmental stress conditions (Siddikee et al., 2010; Jha et al., 2012).

All of the isolates significantly increased the growth of inoculated peanut seedlings under non-stress conditions. The synthesis of phytohormone IAA is a frequently used mechanism of PGPR to enhance plant growth (Dimkpa et al., 2009; Glick, 2012). IAA regulates several aspects of growth and development by controlling critical biological process, such as lateral root initiation, cell enlargement, cell division and increase root surface area that helps in an uptake of soil nutrients (Zhao, 2010). All bacterial isolates, studied here, produce a significant quantity of IAA and their levels are similar or, even, higher than other PGPR that promote growth in various crop species (Karnwal, 2009; Majeed et al., 2015; Zahid et al., 2015).

Nitrogen fixation also contributes to plant growth promotion. For example, a recent study has provided direct evidence through radiolabelling experiment that bacteria (*Herbaspirillum seropedicae* and *A*. *brasilense*) inoculated plants incorporate biologically fixed N in major metabolism processes to promote the plant growth (Pankievicz et al., 2015). All bacterial isolates of the present study reduced acetylene, indicating that they are capable of fixing N. In accordance, a significant increase in the N content in inoculated plants grown in N-free medium was observed. This indicates active biological N fixation is well achieved in the presence of our isolates. Similar observations have also been reported in several plant species previously (Malik et al., 1997; Requena et al., 1997; Figueiredo et al., 2008; Majeed et al., 2015; Pankievicz et al., 2015). These observations imply that the increase in N content and modulation of IAA contents may contribute to peanut growth promotion by PGPR.

The present study also revealed that PGPR isolates enhance salt tolerance of peanut seedlings. Among several mechanisms used by PGPR, maintenance of low Na^+^/K^+^ is considered as a predominant mechanism that favors plant growth under high salinity (Munns and Tester, 2008). In the present study, peanut seedlings treated with three isolates (MBE01, MBE02, and MBE05) showed lower shoot Na^+^/K^+^ ratio than non-inoculated seedlings under salinity stress. These results were in accordance with previous studies where bacterial inoculum has been shown to maintain low Na^+^/K^+^ ratio in various crop plants to reduce salt toxicity (Ozawa et al., 2007; Bano and Fatima, 2009; Shukla et al., 2012; Ramadoss et al., 2013).

Two bacterial isolates (MBE03 and MBE04) enhanced peanut growth under salinity stress; however, no change in shoot Na^+^/K^+^ ratio was observed. Similar observation was previously reported for inoculated tomato (Mayak et al., 2004a). This observation suggested that the strains use alternative mechanisms to favor the plant growth under salinity. In the present study, four isolates including MBE03, and MBE04 had increased shoot Ca^+^ accumulation than non-inoculated control. Ca^+^ is an important secondary molecule that plays a vital role in salt signaling (Kader and Lindberg, 2010). Maintenance of high Ca^+^ levels is a potential mechanism to reduce the damage caused by salt stress (Yang et al., 2016). The PGPR may stimulate plants to selectively take up Ca^+^ to maintain a high Ca^+^/Na^+^ ratio.

Salinity stress leads to excess ROS production and cellular toxicity in plants (Munns and Tester, 2008). The counteract severe effect of oxidative stress plants activate their antioxidant defense machinery that scavenges the excess ROS and maintain redox homeostasis (Munns and Tester, 2008; Tiwari et al., 2013). In the present study, PGPR inoculated seedlings accumulated less reactive oxygen species and affected transcript levels of antioxidant genes. The observation implies that the differential expression of antioxidant genes might be involved in the regulation of ROS level in PGPR treated seedlings under salinity stress. This is in agreement with previous studies where PGPR treatment activated antioxidant defense response, thereby leading to low level accumulation of ROS in plants under salinity stress (Heidari and Golpayegani, 2012; Upadhyay et al., 2012; Gururani et al., 2013). However, we cannot rule out the possibility of other mechanisms rhizobacteria may use to control excess ROS accumulation in inoculated peanut seedlings under salinity stress. In summary, our present results have shown that selective ion uptake and redox homeostasis is an important protective mechanism that PGPR may use to confer salinity stress tolerance in peanut.

Overall, the present study characterized potential halotolerant PGPR that are attributed with several traits related to plant growth promotion, efficiently colonize the roots, increase total plant N and promotes peanut growth under controlled condition. Peanut inoculated with bacterial isolates maintain ion homeostasis and ROS levels under salt stress condition. Future studies focusing on characterization of these strains under multi-location field trials are in progress to commercialize them as biofertilizers.

## Author contributions

SS performed research, design the experiments, analyze data and wrote the paper; JK performed research and help in data analysis; BJ conceived the research and wrote the paper.

### Conflict of interest statement

The authors declare that the research was conducted in the absence of any commercial or financial relationships that could be construed as a potential conflict of interest.
