# Supplementary figures and images for: Halotolerant Rhizobacteria Promote Growth and Enhance Salinity Tolerance in Peanut
Source: Front Microbiol. 2016 Oct 13;7:1600. doi: 10.3389/fmicb.2016.01600 (PMC5062030; doi:10.3389/fmicb.2016.01600)

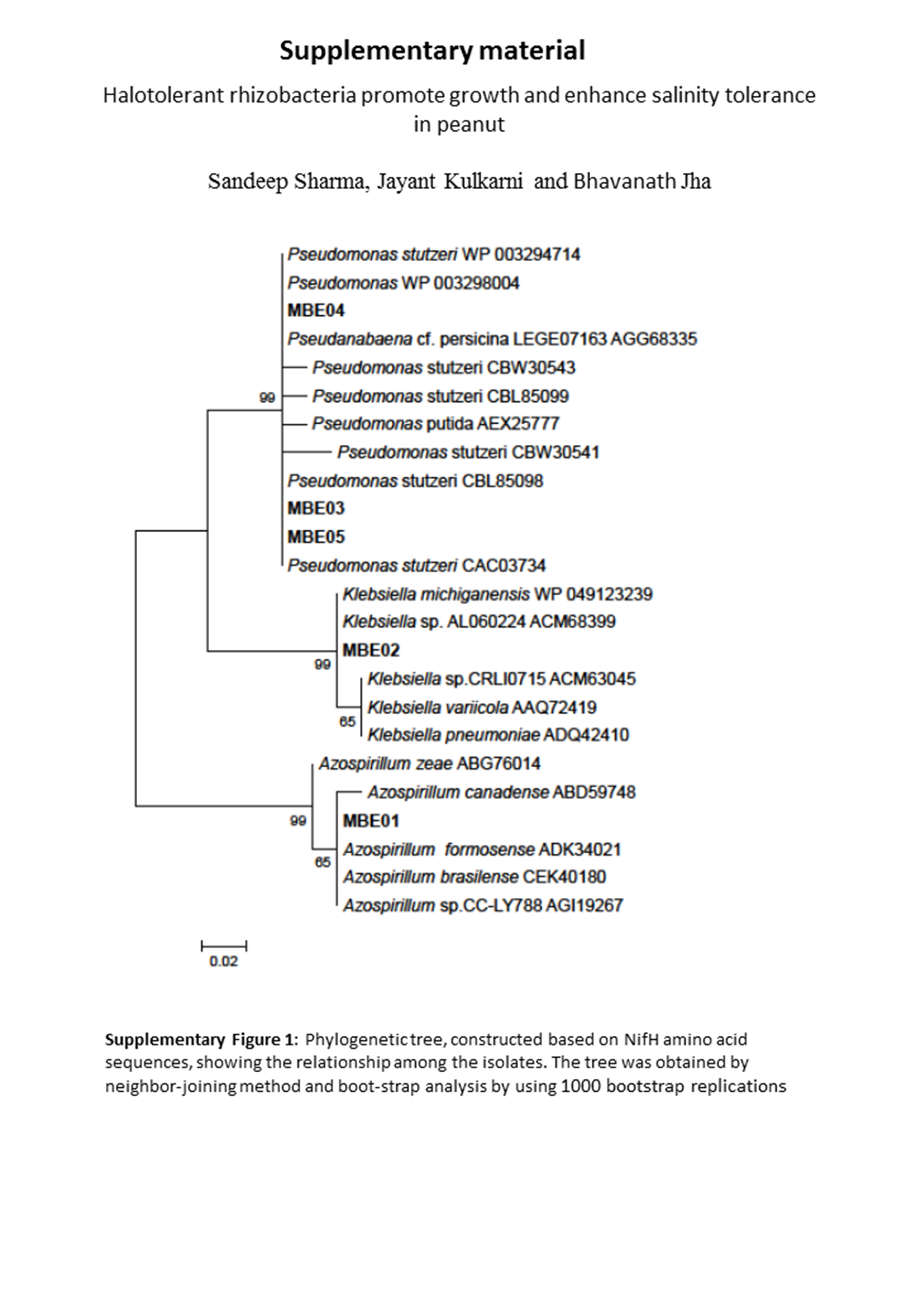

Supplement: Supplementary file 5 [file Image1.TIF]

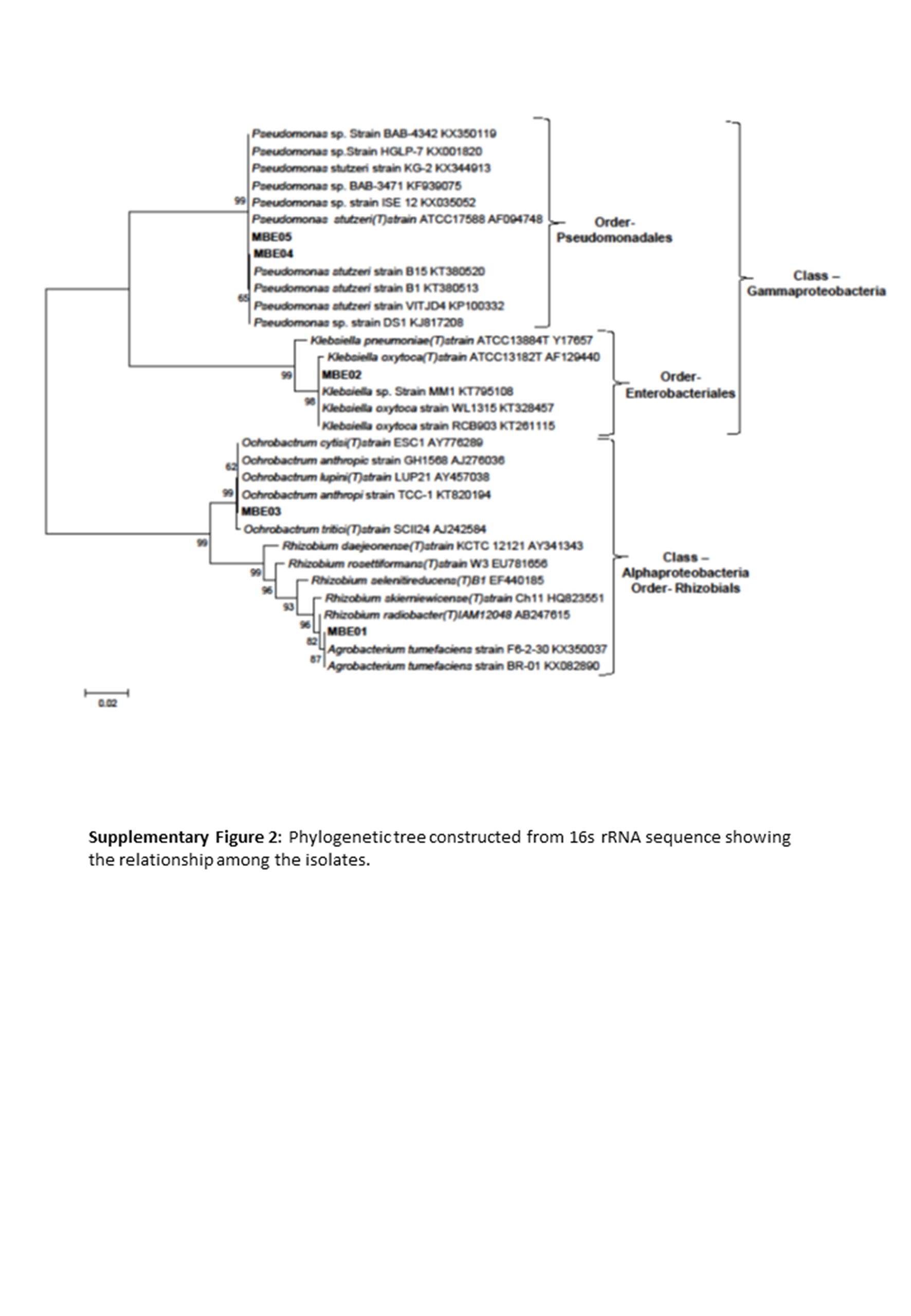

Supplement: Supplementary file 6 [file Image2.TIF]

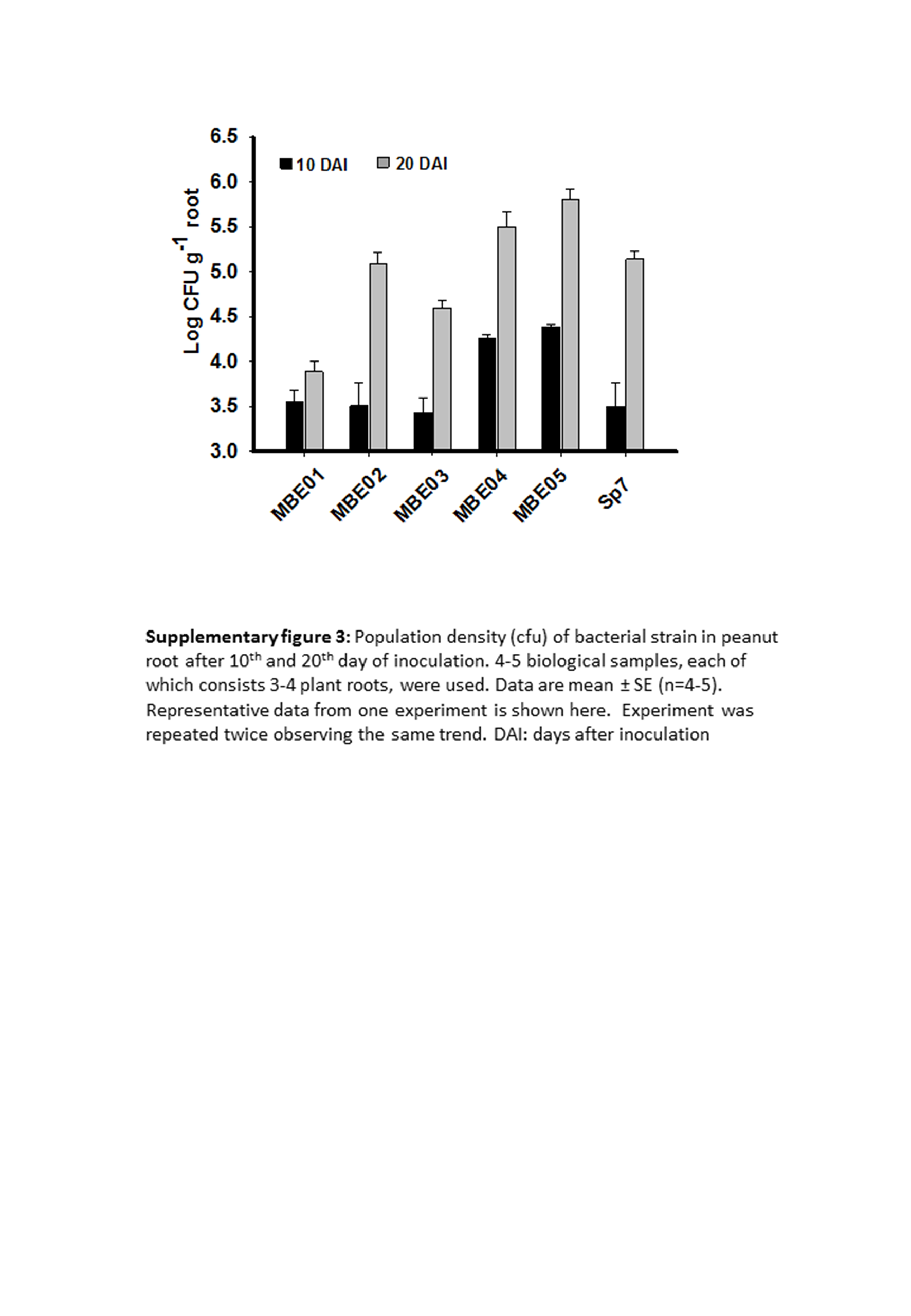

Supplement: Supplementary file 7 [file Image3.TIF]

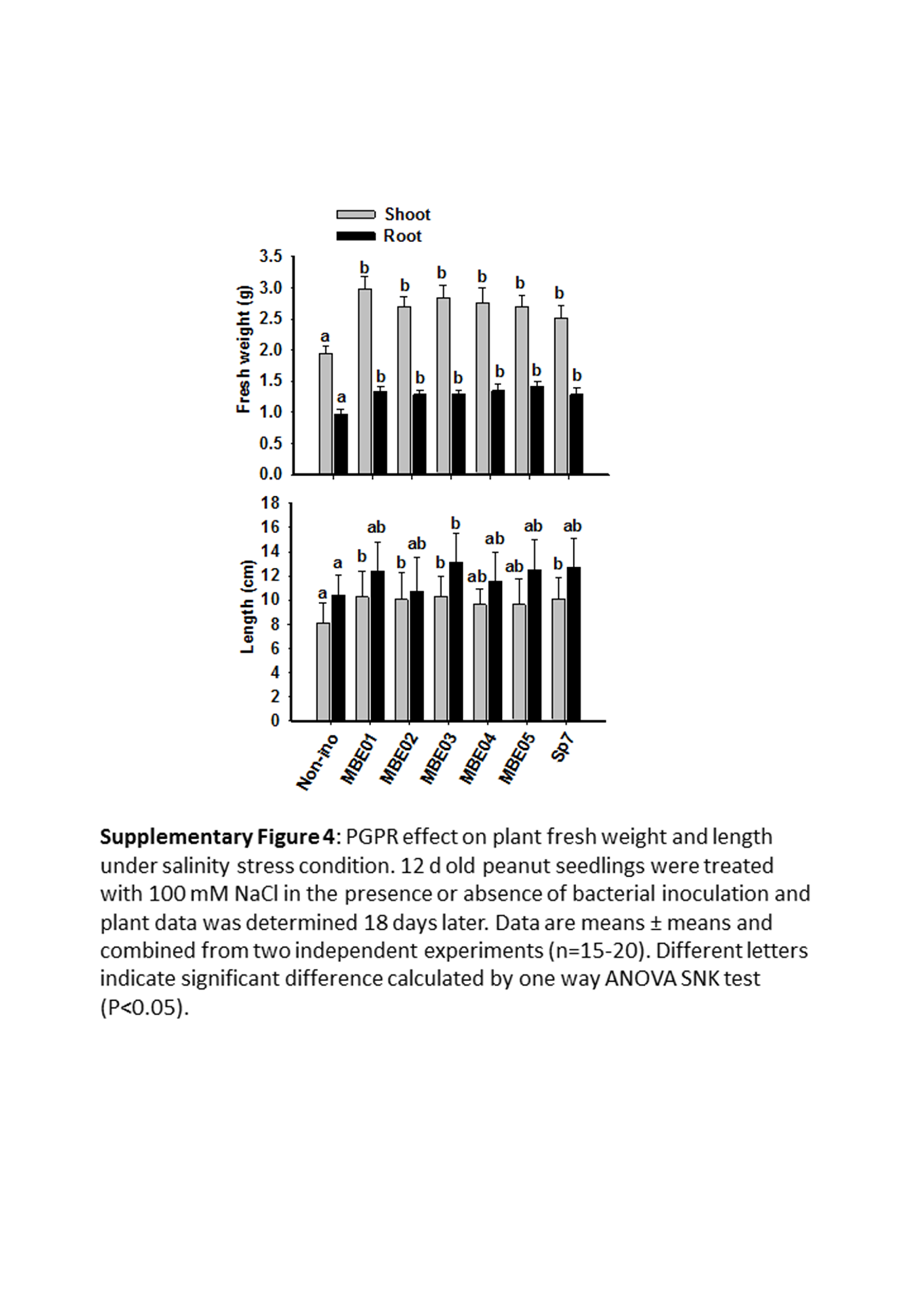

Supplement: Supplementary file 8 [file Image4.TIF]
